# Supplementary material for: Comprehensive identification and expression analysis of CAMTA gene family in Phyllostachys edulis under abiotic stress
Source: PeerJ. 2023 May 8;11:e15358. doi: 10.7717/peerj.15358 (PMC10174056; doi:10.7717/peerj.15358)
Supplement: Supplemental Information 4 — Cold stress treatment; Heat stress treatment; Drought stress treatment. [file peerj-11-15358-s004.pdf]

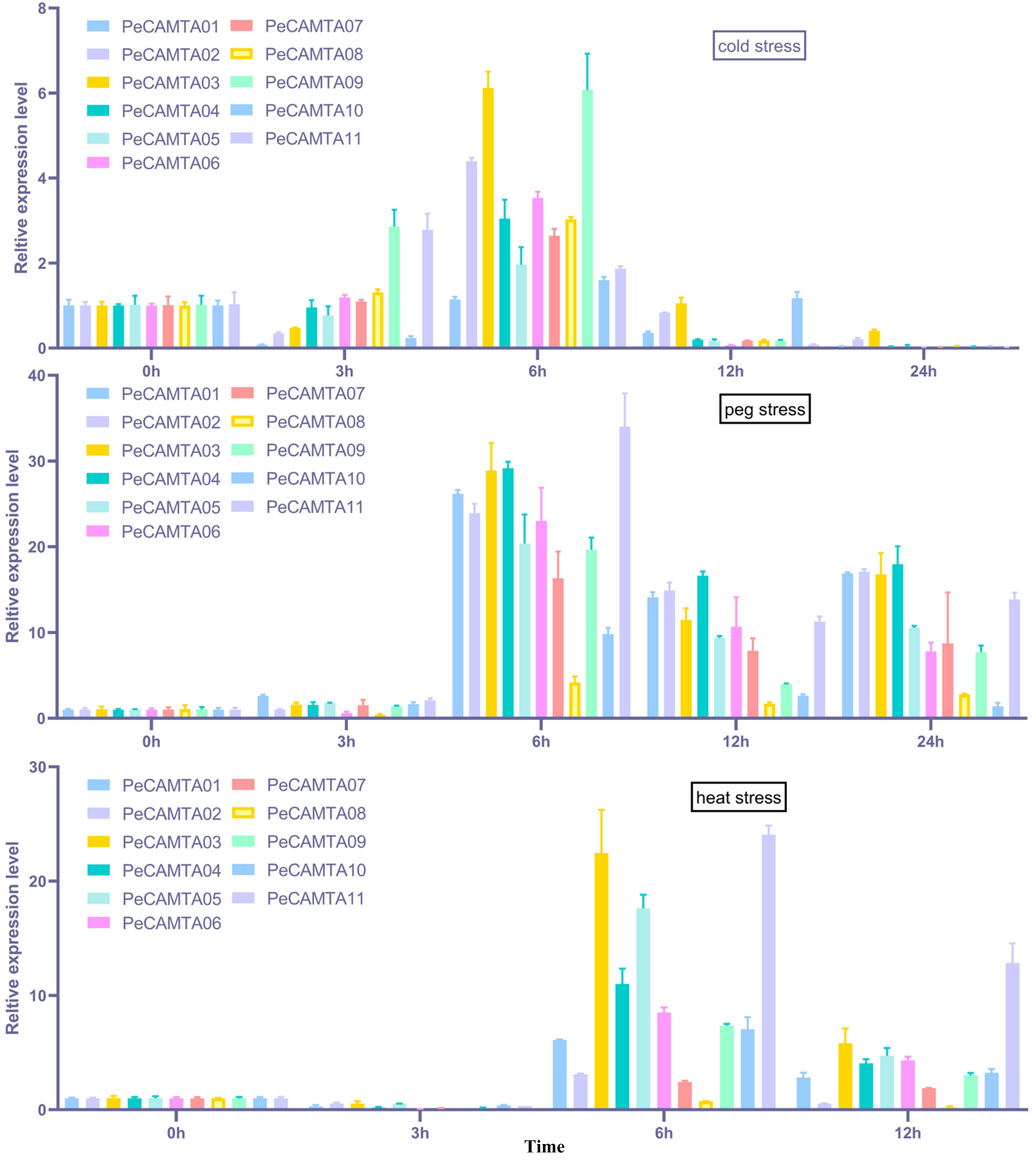

cold stress
peg stress
heat stress
PeCAMTA01
PeCAMTA02
PeCAMTA03
PeCAMTA04
PeCAMTA05
PeCAMTA06
PeCAMTA07
PeCAMTA08
PeCAMTA09
PeCAMTA10
PeCAMTA11
Reltive expression level
0h
3h
6h
12h
24h
Time
